# NRF2 Activation in Autophagy Defects Suppresses a Pharmacological Transactivation of the Nuclear Receptor FXR

**DOI:** 10.3390/antiox11020370

**Published:** 2022-02-12

**Authors:** Eun Young Kim, Jae Man Lee

**Affiliations:** 1 Department of Biochemistry and Cell Biology, Cell and Matrix Research Institute, School of Medicine, Kyungpook National University, Daegu 41944, Korea; key11@knu.ac.kr; 2 BK21 FOUR KNU Biomedical Convergence Program, Department of Biomedical Science, The Graduate School, Kyungpook National University, Daegu 41944, Korea

**Keywords:** autophagy, nrf2, keap1, nuclear receptor, FXR, transactivation, ligand

## Abstract

NF-E2-related factor 2 (NRF2), an antioxidant transcription factor, is activated in autophagy-deficient mice due to the accumulations of p62/SQSTM1 and its subsequent interaction with Kelch-like-ECH-associated protein 1 (KEAP1), an adaptor component for Cullin3-based E3 ubiquitin ligase complex. Farnesoid x receptor (FXR/NR1H4) is a ligand-dependent transcription factor that belongs to the nuclear receptor superfamily. FXR plays an essential role in bile acid synthesis and enterohepatic circulation, affecting glucose and lipid metabolism. Obeticholic acid as a potent FXR agonist has been approved to treat primary biliary cholangitis and clinical trials for its use in the treatment of other liver diseases are underway. Here we show that NRF2 activation in autophagy defects impedes a transactivation of FXR. Liver-specific *Atg7* knockout mice or a treatment of autophagy inhibitor showed decreased inductions of FXR target genes upon its synthetic agonists. Moreover, enforced NRF2 activations with small molecules potently decreased the pharmacological activation of FXR in cultured cells. Finally, we demonstrate that NRF2 activation by the treatment with the food antioxidant butylated hydroxyanisole is necessary and sufficient to inhibit the pharmacological activation of FXR in vivo. These results reveal a novel function of the basal autophagy-NRF2 axis for the regulation of FXR transactivation, and shed light on a potential therapeutic strategy in metabolic disease.

## 1. Introduction

NRF2 is a member of the “cap’n’collar” (CNC) family of DNA-binding transcription factors, featured by a conserved basic region-leucine zipper (bZIP) motif, CNC domain, and transactivation/transrepression domain [1,2]. The transcriptional activation of NRF2 requires a heterodimerization with a member of the small Maf protein family, allowing them to bind to the antioxidant or electrophile responsive element (ARE/EpRE) [3]. NRF2 controls the transcriptional programs of a series of phase 2 detoxifying genes and phase 3 transporters via ARE sites within their regulatory regions [4,5]. Moreover, its physiological significance as defense mechanisms against xenobiotics has been demonstrated by the analyses of *Nrf2* knockout mice that are susceptible to the challenges of toxic insults including acetaminophen [6,7], diesel exhaust [8], and antioxidant butylated hydroxytoluene [9]. The roles of NRF2 in inflammatory and metabolic disease and aging have recently been reviewed [10,11,12].

A yeast-two hybrid screen isolated the Kelch-like ECH-associated protein 1 (KEAP1) as an NRF2 interaction protein. As an adaptor protein for the Cullin3-based E3 ubiquitin ligase complex, KEAP1 promotes the degradation of NRF2 under unstressed conditions [13,14]. However, as an oxidative stress sensor in cells, KEAP1 thiols are modified upon the exposure of redox-disrupting stimuli such as electrophiles, liberating NRF2 from the ubiqutin-mediated proteasomal degradation system. This leads to NRF2 accumulation in the nucleus where it increases ARE-mediated gene expression [15].

Autophagy is the cellular process by which cells deliver their own proteins, lipids, and organelles to the lysosome [16]. Autophagy typically involves the formation of double membrane-bound vesicles, so-called autophagosomes, encompassing cytoplasmic molecules, bacteria, and organelles as cargos. Autophagosomes are then fused with lysosomes to become autolysosomes where these cargo molecules are broken down [17]. The degraded materials are then released from the lysosome to the cytoplasm where they are recycled for the generation of energy and the formation of new macromolecules to sustain survival [18]. Autophagy activity can be robustly increased upon stress conditions such as nutrient deprivation and pathogen infection, and is required for these stress adaptations, although most cells have a basal autophagy activity under unstressed conditions [16]. Autophagy also plays a key role in the maintenance of organelle function and tissue homeostasis by preventing the accumulation of toxic waste products, and thereby sustaining cellular metabolism and survival during fasting periods [19]. Autophagy defects trigger the inability to remove unfolded proteins and toxic materials, resulting in the accumulation of p62/SQSTM1 and damaged mitochondria. These lead to the generation of reactive oxygen species and subsequent damages to the genome as well as endoplasmic reticulum stress [20,21]. These autophagy functions have been known to be genetically programmed by autophagy-related genes and their regulatory genes that enable initiation of autophagy, recognition of cargos, formation of autophagosome, and autolysosome [22].

Tissue-specific ablations of core-autophagy gene *Atg5* or *Atg7* using a Cre-loxP system have unveiled that macroautophagy is critical for maintaining tissue homeostasis. It has been also shown that liver-specific *Atg7* (*Atg7^LKO^*) knockout mice showed hepatomegaly phenotypes and marked accumulations of p62, a ubiquitin-binding protein that acts as an autophagy adaptor protein. Increased levels of p62 can interact with KEAP1, an adaptor protein of Cullin3-based E3 ubiquitin ligase complex, which liberates NRF2 from ubiquitin-mediated proteasomal degradation system. As a potent antioxidant transcription factor, the nuclear NRF2 binds to the ARE of many cytoprotective genes such as *NAD(P)H dehydrogenase, quinone 1* (*Nqo1*), *Heme oxygenase 1* (*Hmox-1*), *Glutathione S-transferase alpha 1 (Ya)* (*Gsta1*), and so forth, and upregulates their expressions to remove reactive oxygen species.

Among 48 members of nuclear receptors in the human genome, FXR is one of the most notably adopted orphan nuclear receptors due to the discoveries of a few bile acids as endogenous ligands [23,24,25,26,27,28]. Hepatic FXR has been known to be typically activated by bile acids returning to the liver along with fat soluble nutrients in the process of enterohepatic circulation in the fed state, and to coordinate expression of genes involved in bile acid homeostasis and its associated metabolism [29,30,31]. Several natural and synthetic ligands have been identified or developed that have potential for clinical applications [32,33,34,35,36,37]. FXR activation using obeticholic acid (OCA) has been already approved for the treatment of primary biliary cholangitis and is currently underway for the treatment of nonalcoholic steatohepatitis [38,39]. Consistent with its essential roles in feeding conditions, previous studies have been shown that FXR is necessary for suppressing autophagy in the fed state, and that pharmacological activation of FXR is sufficient for suppressing autophagy even in the fasted state. This autophagy suppression by FXR seems to occur at the transcription levels via downregulation of core-autophagy-related genes. Molecular mechanisms for this have been shown that FXR directly competes with peroxisome proliferator-activated receptor alpha (PPARα), a fasting-activated nuclear receptor to bind to a direct repeat 1 in the regulatory regions of core-autophagy-related genes [40]. Alternatively, FXR enables a disputation of the cAMP response element-binding protein (CREB)-CREB regulated transcription coactivator 2 (CRTC2) complex critical for the upregulation of autophagy genes [41]. A recent study has suggested a novel mechanism by which FXR activation impairs autophagy in models of human cholestasis via the induction of the gene encoding a RUN domain and cysteine-rich domain containing, Beclin 1-interacting protein (Rubicon) as a direct FXR target gene. The FXR-mediated RUBICON complex seems to inhibit the final fusion process between autophagosome and lysosome to make autolysosome [42].

In this study, we want to define whether NRF2 activation in autophagy defects affects functionalities of the nuclear bile acid receptor FXR. We find that hepatic autophagy inhibition in *Atg7^LKO^* mice and in cultured cells blunts a pharmacological induction of FXR target genes. Furthermore, dose- or time-dependent treatments of NRF2 activators are able to potently inhibit the pharmacological activation of FXR in AML12 cells. Finally, we have demonstrated that upon butylated hydroxyanisole (BHA) treatment, NRF2 is necessary and sufficient to impede the pharmacological activation of FXR. Our results shed light on the mechanisms of impaired activation of FXR by the NRF2 activation in autophagy defects. Because impaired autophagy often occurs in metabolic disorders, these findings may provide a potential therapeutic strategy to treat chronic liver disease.

## 2. Materials and Methods

### 2.1. Chemicals and Reagents

Wild-type C57BL/6J mice were purchased from Japan SLC, Inc. (Japan) (C57BL/6JJmsSlc); *Atg7^F/F^* (RBRC02759) [43] mice were purchased from RIKEN BioResource Research Center (Japan); *Alb-Cre* mice (strain #003574) [44] and *Nrf2^F/F^* mice (strain #025433) [45,46] were purchased from the Jackson laboratory (USA); *Keap1^F/F^* mice [47] were obtained from the Masayuki Yamamoto laboratory. AML12 cells were purchased from ATCC (USA) (CRL-2254); GW4064 (Cat.# 2473) from Tocris (UK); Obeticholic acid (OCA, Cat.# AG-CR1-3560-M025) from Adipgen (USA); L-sulforaphane (SFN, Cat.# 14797) from Cayman (USA); Dimethyl fumarate (DMF, Cat.# 242926), butylated hydroxyanisole (BHA, Cat.# B1253-500G), dexamethasone (Cat.# D4902), polyethylene glycol 400 (PEG 400, Cat.# P3265-1KG), Tween 80 (Cat.# P1754-500ML), trizma phosphate (Cat.# T-8655), adenosine triphosphate (ATP, Cat.# A7699), magnesium chloride (Cat.# 208337), and dipotassium phosphate (K_2_HPO_4_, Cat.# P3786) were purchased from Sigma-Aldrich (USA); Bafilomycin A1 (BafA1, Cat.# BML-CM110) from Enzo Life Science (USA); HyClone DMEM/F12 (Cat.# SH30023.01) and fetal bovine serum (FBS, Cat.# SH20084.03) from HyClone (USA); penicillin-streptomycin (Cat.# 15140122) from Gibco (USA); Insulin-Transferrin-Sodium Selenite Supplement (ITS, Cat.#11074547001) from Roche (Switzerland); Trizol Reagent (Cat.# 15596018) from Invitrogen (USA); RbTaq^TM^ qPCR 2× PreMIX (SYBR Green with high ROX, Cat.# RT531M) from Enzynomics (Korea); PrimeScript^TM^ 1st strand cDNA Synthesis kit (Cat.# 6110A) from TaKaRa (Japan); Dimethyl sulfoxide (DMSO, Cat.# sc-358801) from Santa Cruz; galacton-plus substrate I 100 X concentrate (Cat.# T218) from Applied Biosystems (USA); ACCELERATOR-II 210 ML (Cat.# T2222) from Tropix (USA); D-Luciferin (Cat.# L-8240) from Biosynth Carbosynth (UK). Information for other reagents not shown here is described in the relevant methods and references sections.

### 2.2. Animal Studies

All animal studies and procedures were approved by the institutional Animal Care and Use Committee of the Kyungpook National University (KNU-2020-036). Wild-type mice were C57BL/6J mice. Male *Alb-Cre/+* mice were bred with either female *Atg7^F/F^*, *Keap1^F/F^*, or *Nrf2^F/F^* mice to generate *Alb-Cre*; *Atg7^F/F^* (*Atg7^LKO^*), *Alb-Cre*; *Keap1^F/F^* (*Keap1^LKO^*), or *Alb-Cre*; *Nrf2^F/F^* (*Nrf2^LKO^*) mice, which showed a hepatocyte-specific ablation of either *Atg7*, *Keap1*, or *Nrf2* gene, respectively. All experiments were performed in ad libitum-fed male mice unless otherwise indicated. To activate PPARα in the liver, 8–9 week-old male wild-type mice C57BL/6J, *Atg7^F/F^*, *Atg7^LKO^*, *Alb-Cre/+*, *Keap1^LKO^*, *Nrf2^F/F^*, or *Nrf2^LKO^* mice were intraperitoneally injected with vehicle (0.1% dimethylsulfoxide (DMSO) in 90:5:5 of saline, PEG-400 and Tween 80, respectively) or GW4064 (100 mg/kg BW) twice a day (first injection at 00:00 and second injection at 12:00). The mice were sacrificed 5 h after the second injection, to collect the livers. To activate NRF2, 8–9 week-old male wild-type C57BL/6J, *Nrf2^F/F^*, or *Nrf2^LKO^* mice were orally gavaged with either the vehicle or butylated hydroxyanisole (BHA, 200 mg/kg BW) once a day at 12:00 for 3 days. To avoid circadian issues, all mice were sacrificed at 17:00–18:00.

### 2.3. Cell Culture and Drug Treatments

**Figure 1:**
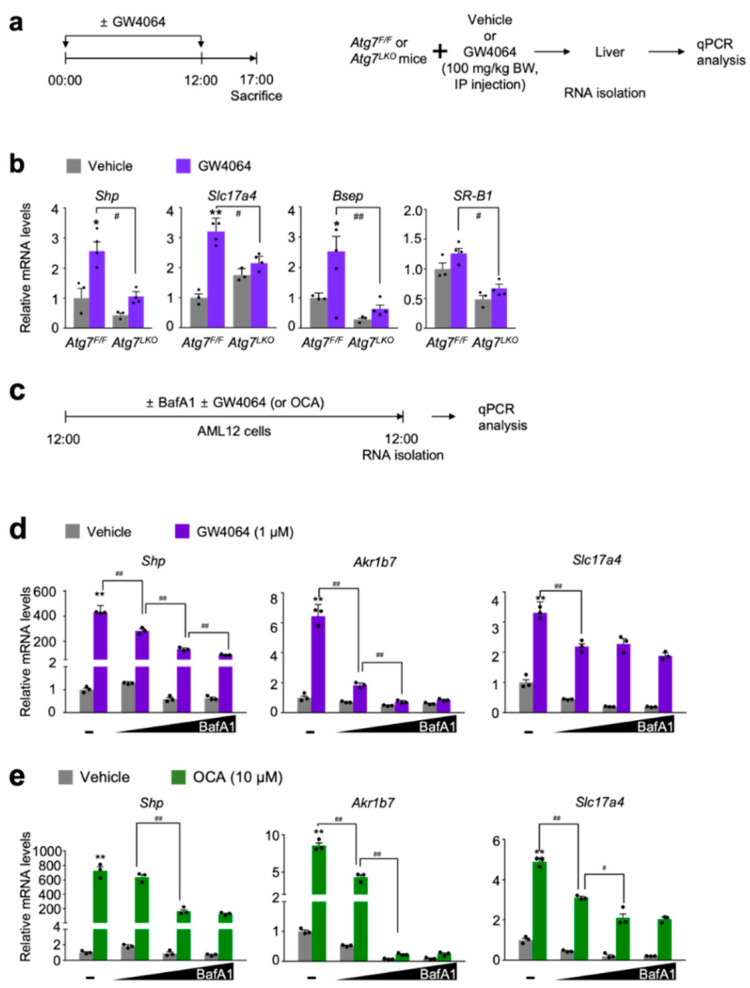
Autophagy inhibition impairs a pharmacological transactivation of FXR. (**a**) A schematic diagram of an experimental procedure in mice. 8- to 9-week-old male *Atg7^F/F^* and *Atg7^LKO^* mice were intraperitoneally injected with vehicle (0.1% DMSO in 4:1 ratio of PEG 400 and Tween 80) or GW4064, a synthetic FXR agonist (100 mg/kg BW) twice a day. 5 h after last injection, all mice were sacrificed to harvest livers whose total RNAs were isolated for qPCR analysis; (**b**) Hepatic expression levels of FXR target genes *Shp*, *Slc17a4*, *Bsep*, and *SR-B1* were determined in *Atg7^F/F^* and *Atg7^LKO^* mice shown in panel (**a**) by qPCR analysis. *n* = 3–4 per group, * *p* < 0.05, ** *p* < 0.01 vs. *Atg7^F/F^* mice treated with vehicle. ^#^ *p* < 0.05, ^##^ *p* < 0.01. Data represent mean ± s.e.m. and are plotted as fold change. Each dot indicates individual mouse. Statistics by two-tailed *t*-test; (**c**) A schematic diagram of an experimental procedure in AML12 cells, a mouse hepatocyte-derived cell line. AML12 cells were treated with bafilomycin A1 (BafA1: 0.1, 0.5, or 1 μM), a known autophagy inhibitor in the absence or presence of synthetic FXR agonists GW4064 (1 μM) or obeticholic acid (OCA, 10 μM) for 24 h. Vehicle is 0.1% DMSO. Total RNAs from these cells were prepared to perform qPCR analysis; (**d**,**e**) Expression levels of FXR target genes *Shp*, *Akr1b7*, and *Slc17a4* were determined in AML12 cells shown in panel (**c**) by qPCR analysis. *n* = 3 per group; ** *p* < 0.01 vs. AML12 cells treated with vehicle; ^#^ *p* < 0.05; ^##^ *p* < 0.01. Data represent mean ± s.e.m. and are plotted as fold change. Each dot indicates individual sample. Statistics by a two-tailed, unpaired Student *t*-test. BW, body weight.

AML12 cells were maintained in the complete media: DMEM/F12 high glucose supplemented with 10% FBS, 1% ITS, 1% penicillin-streptomycin antibiotics and 40 ng/mL dexamethasone. About 70–80% confluent cells were seeded in a 6- or 12-well plate in a 1:5 ratio 48 h before drug treatments. To assess a pharmacological activity of FXR in an autophagy-inhibited condition (Figure 1c–e and Figure S2c–e), these cells were treated with bafilomycin A1 (BafA1) in a dose-dependent manner (BafA1, 0.1, 0.5, or 1 μM). Simultaneously, they were also treated with synthetic FXR agonists 1 μ GW4064 or 10 μM OCA. To determine a pharmacological activity of FXR in NRF2-activated conditions, AML12 cells were treated with either sulforaphane (SFN, 12.5, 25, or 50 μM) or dimethyl fumarate (DMF, 50, 75, or 100 μM) in a dose-dependent manner. Likewise, these cells were also dose-dependently treated with GW4064 (0.1, 0.5, or 1 μM) or OCA (1, 5, or 10 μM). On the other hand, AML12 cells were treated with either sulforaphane (25 μM SFN, 6, 12, or 18 h) or dimethyl fumarate (75 or 100 μM DMF, 6, 12, or 18 h) in a time-dependent manner. These cells were simultaneously treated with 1 μM GW4064 or 10 μM OCA for 24 h. The vehicle was 0.1% DMSO. Mouse embryonic fibroblasts derived from wild-type, *Atg5^−/−^*, or *Atg7^−/−^* mouse embryos were maintained in DMEM high glucose supplemented with 10% FBS, 1% ITS, and 1% penicillin-streptomycin antibiotics.

### 2.4. RNA Purification, cDNA Synthesis and qPCR Analysis

Total RNA was purified from the snap-frozen mouse liver or AML12 cells using Trizol Reagent according to the manufacturer’s instructions (Invitrogen). The RNA concentration and quality was determined by Nanodrop. 1 μg of total RNA was converted to cDNA using PrimeScript^TM^ 1st strand cDNA Synthesis kit (Takara). The qPCR was performed with RbTaq^TM^ qPCR 2× PreMIX (SYBR Green with high ROX, Enzynomics) on StepOnePlus Real-Time PCR systems (Applied Biosystems). All reactions were carried out in either duplicate or triplicate and C_t_ values were obtained. The mRNA levels were normalized to the expression levels of the housekeeping gene 36b4 (also known as Rplp0) using the standard curve method. Information of mouse primer sequences for qPCR analysis is listed in Table S1.

### 2.5. Cell-Based Reporter Assay

AML12 cells were seeded in 24-well plates. Transient transfections were performed using Lipofectamine 2000. These cells were transfected with 200 ng of 2× *PLTP*-pTK-Luc reporter construct, 100 ng of cytomegalovirus-promoter (CMX)-human FXR or CMX-human retinoic acid receptor alpha (RXRα), and 50 ng of CMX-β-galactosidase. pCDNA3.1 plasmid was added to prepare the total DNA to 500 ng per well. After 24 h transfection, cells were treated with drugs (GW4064, 1 μM; 9-cis retinoic acid (9-cis RA), 1 μM; obeticholic acid (OCA), 10 μM) in the presence or absence of sulforaphane (SFN, 12.5, 25, or 50 μM) in a dose-dependent manner for 24 h before performing luciferase and β-galactosidase assay. Luciferase activity was normalized with β-galactosidase activity. Normalized values from vehicle-treated cells were set as fold 1. Each dot indicates each experiment consisting of 3-wells per group.

### 2.6. Statistical Analysis

All values are shown as mean ± s.e.m. and error bars were derived from biological replicates rather than technical replicates. Significant differences between two groups were evaluated using a two-tailed, unpaired Student *t*-test, which was found to be appropriate as groups displayed a normal distribution and comparable variance, *p* < 0.05 was considered statistically significant.

## 3. Results

### 3.1. Autophagy Defect Impairs a Pharmacologic Activation of FXR

To define whether autophagy defects affect nuclear receptor signaling, liver-specific *Atg7* ablation (*Atg7^LKO^*) in mice were produced by crossing female *Atg7^F/F^* mice with male *Alb-Cre* mice. Consistent with previous literatures, the livers of *Atg7^LKO^* mice showed hepatomegaly phenotypes compared with those of control littermates *Atg7^F/F^* mice (data not shown). As liver-specific knockouts, hepatic mRNA and protein levels of *Atg7* gene were also markedly decreased in *Atg7^LKO^* mice (data not shown).

A previous study has shown that serum levels of β-hydroxybutyrate in control *Atg7^F/F^* mice were elevated during fasting or upon treatment of a synthetic PPARα agonist GW7647 but these responses were compromised in *Atg7^LKO^* mice [40], allowing us to have a hypothesis that core-autophagy-related genes play a pivotal role in functionalities of at least some of nuclear receptors, including FXR.

To test this hypothesis, hepatic expression levels of FXR target genes *Shp* (also known as *Nr0b2*), *Slc17a4*, *Bsep* (also known as *Abcb11*), and *SR-B1* (also known as *Scarb1*) were determined in both *Atg7^F/F^* control littermates and *Atg7^LKO^* mice in response to the intraperitoneal treatment of GW4064 (Figure 1a). As expected, pharmacological inductions of these hepatic FXR target genes were observed in *Atg7^F/F^* mice compared with those of vehicle treated counterparts. However, these inductions were markedly decreased in GW4064-treated *Atg7^LKO^* mice (Figure 1b).

To investigate whether similar results could occur in cultured mammalian cells, mouse hepatocyte-derived cell-line AML12 cells were dose-dependently treated with bafilomycin A1 (BafA1), an autophagy inhibitor, inactivating lysosomal V-ATPase enzyme [48,49,50]. At the same time, these cells were co-treated with or without synthetic FXR agonist ligands GW4064 [32] or obeticholic acid (OCA, also known as INT-747 or 6α-ethyl-chenodeoxycholic acid) [33] (Figure 1c). Consistent with the in vivo mouse data shown in Figure 1b, dose-dependent treatments of bafilomycin A1 potently blunted inductions of FXR target genes *Shp*, *Akr1b7*, and *Slc17a4* in response to the treatment of these synthetic agonist ligands (Figure 1d,e). All these results from in vivo mouse and cell culture experiments demonstrate that both genetic and pharmacological inhibitions of autophagy impair synthetic agonist effects of FXR.

It has been reported that p62 (also known as SQSTM1), a ubiquitin-binding protein, was markedly accumulated by genetic deletions of core-autophagy-related genes such as *Atg7* [51]. This overproduction of p62 in the context of autophagy defects promotes its interaction with the NRF2-binding sites on KEAP1, a component of Cullin3-type E3 ubiquitin ligase. This then leads to stabilization and subsequent nuclear translocation of NRF2, resulting in the induction of its target genes, including phase II cytoprotective genes [52]. As previously reported, we also found a profound induction of the *Nqo1* gene, a NRF2 target gene in the livers of *Atg7^LKO^* mice compared with those of *Atg7^F/F^* mice (Figure S1a). Based on these data, we concluded that our *Atg7^LKO^* mice significantly increased hepatic NRF2 activation. It is of interest to note that the pharmacological activation of FXR in response to GW4064 treatment does not alter hepatic *Nqo1* expression compared with vehicle-treated counterparts (Figure S1a), indicating that FXR activation may not affect NRF2 transactivation. In addition, a dose-dependent treatment of bafilomycin A1 in AML12 cells significantly increased the induction of *Hmox-1* gene, another well-established NRF2 target gene, but gradually decreased *Nqo1* expression (Figure S1b,c), demonstrating a differentially regulated expression kinetics of two NRF2 target genes in the autophagy defective condition.

### 3.2. Pharmacological Transactivation of FXR Is Compromised by the Treatment of NRF2 Activators in a Dose-Dependent Manner

**Figure 2:**
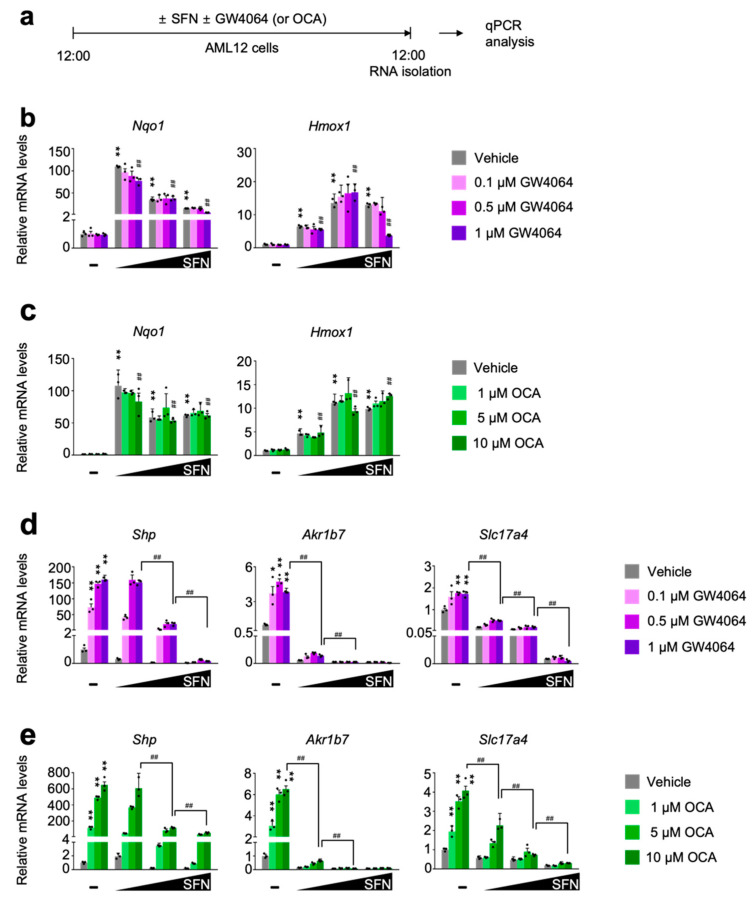
Sulforaphane treatment in a dose-dependent manner impairs a pharmacological transactivation of FXR. (**a**) A schematic diagram of an experimental procedure in AML12 cells. AML12 cells were treated with sulforaphane (SFN: 12.5, 25, or 50 μM), a small molecule of NRF2 activator in a dose-dependent manner in the absence or presence of synthetic FXR agonists (GW4064: 0.1, 0.5 or 1 μM; OCA: 1, 5 or 10 μM) for 24 h. Vehicle is 0.1% DMSO. Total RNAs from these cells were prepared to perform qPCR analysis; (**b**,**c**) Expression levels of NRF2 target genes *Nqo1* and *Hmox1* were determined in AML12 cells shown in panel (**a**) by qPCR analysis. *n* = 3 per group, ** *p* < 0.01 vs. AML12 cells treated with vehicle. ^##^ *p* < 0.01 vs. AML12 cells treated with 1 μM GW4064. Data represent mean ± s.e.m. and are plotted as fold change. Each dot indicates an individual well; (**d**,**e**) Expression levels of FXR target genes *Shp*, *Ark1b7*, and *Slc17a4* were determined in AML12 cells shown in panel (**a**) by qPCR analysis. *n* = 3 per group; * *p* < 0.05; ** *p* < 0.01 vs. AML12 cells treated with vehicle; ^##^ *p* < 0.01. Data represent mean ± s.e.m. and are plotted as fold change. Each dot indicates individual sample. Statistics by a two-tailed, unpaired Student *t*-test. OCA, obeticholic acid.

To define whether pharmacological activation of NRF2 directly affects FXR transactivation, AML12 cells were dose-dependently treated with either sulforaphane [53,54] or dimethyl fumarate [55], two small molecules for the potent NRF2 activation. In the meantime, these cells were co-treated with or without synthetic FXR agonist ligands GW4064 or OCA (Figure 2a and Figure S2a). As expected, these NRF2 activators robustly increased expression levels of NRF2 target genes *Nqo1* and *Hmox1* that were not altered in general by the treatment of synthetic FXR agonists GW4064 or OCA (Figure 2b,c and Figure S2b,c). These data indicate that pharmacological activation of FXR may not affect sulforaphane or dimethyl fumarate-mediated NRF2 activation. Intriguingly, however, completely opposite results were observed in terms of a pharmacological activation of FXR in the presence of NRF2 activators. We found that dose-dependent treatments of sulforaphane or dimethyl fumarate markedly downregulated expression of FXR target genes *Shp*, *Akr1b7*, and *Slc17a4* in response to GW4064 or OCA treatment (Figure 2d,e and Figure S2d,e). All these data suggest that NRF2 activation in AML12 cells is sufficient to suppress a pharmacologic efficacy of synthetic FXR agonists.

### 3.3. Time-Dependent Treatments of NRF2 Activators Suppress Pharmacological Transactivation of FXR

**Figure 3:**
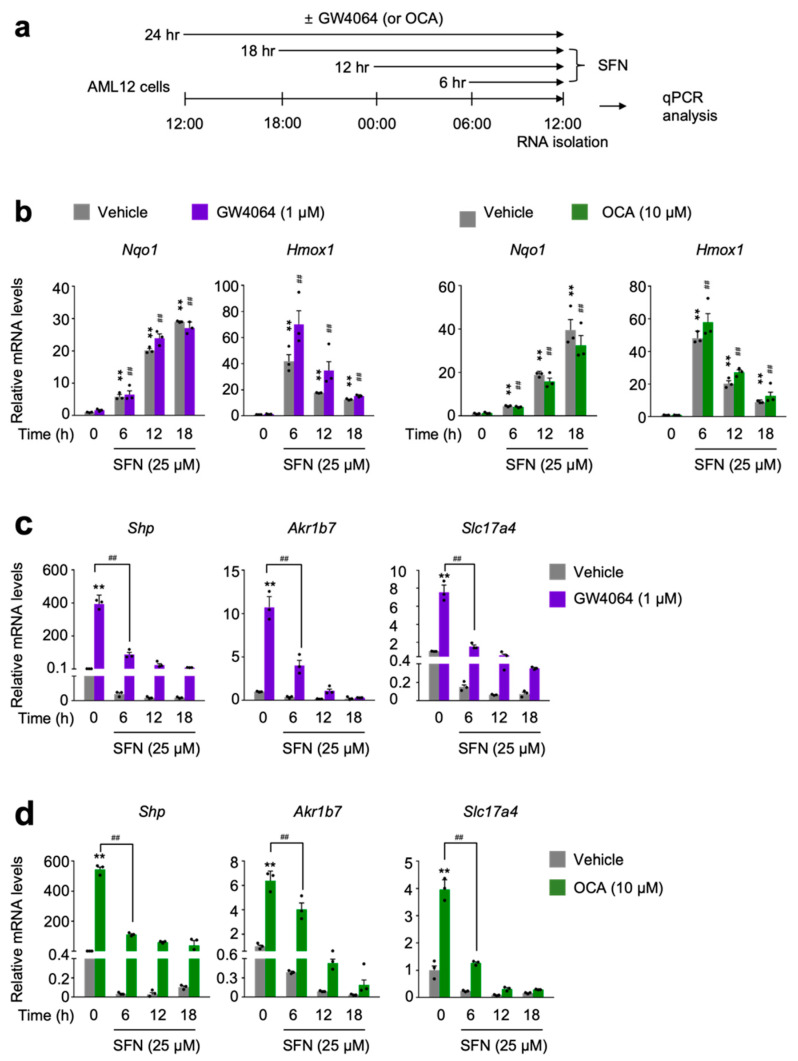
Sulforaphane treatment in a time-dependent manner impairs a pharmacological transactivation of FXR. (**a**) A schematic diagram of an experimental procedure in AML12 cells. AML12 cells were treated with sulforaphane (SFN: 25 μM), a small molecule of NRF2 activator in a time-dependent manner (6 h, 12 h, or 18 h) in the absence or presence of synthetic FXR agonists GW4064 (1 μM) or OCA (10 μM) for 24 h. Vehicle is 0.1% DMSO. Total RNAs from these cells were prepared to perform qPCR analysis; (**b**) Expression levels of NRF2 target genes *Nqo1* and *Hmox1* were determined in AML12 cells shown in panel (**a**) by qPCR analysis. ** *p* < 0.01 vs. AML12 cells treated with vehicle. ^##^ *p* < 0.01 vs. AML12 cells treated with either GW4064 or OCA; (**c**,**d**) Expression levels of FXR target genes *Shp*, *Akr1b7*, and *Slc17a4*) were determined in AML12 cells shown in panel (**a**) by qPCR analysis. *n* = 3 per group; ** *p* < 0.01 vs. AML12 cells treated with vehicle; ^##^ *p* < 0.01. Data represent mean ± s.e.m. and are plotted as fold change. Each dot indicates individual sample. Statistics by a two-tailed, unpaired Student *t*-test. OCA, obeticholic acid.

To further examine whether NRF2 activation directly affects FXR activity, AML12 cells were time-dependently treated with sulforaphane or dimethyl fumarate in the presence or absence of GW4064 or OCA (Figure 3a and Figure S3a). As expected, these NRF2 activators dramatically increased expression levels of *Nqo1* gene in a time-dependent manner (Figure 3b and Figure S3b). However, as shown above, we again found different expression kinetics of two known NRF2 target genes *Nqo1* and *Hmox1*. Six h treatment of NRF2 activators showed the highest expression levels of *Hmox1* gene whose mRNA levels were gradually decreased by increasing the duration time of NRF2 activators (Figure 3b and Figure S3b). Nevertheless, time-dependent treatments of sulforaphane or dimethyl fumarate in AML12 cells dramatically decreased expression of FXR target genes *Shp*, *Akr1b7* and *Slc17a4* in response to GW4064 or OCA (Figure 3c,d and Figure S3c,d). All these data support that acute NRF2 activation is also sufficient for suppressing a pharmacologic FXR activation.

### 3.4. NRF2 Is Necessary and Sufficient for Suppressing a Pharmacologic Transactivation of FXR In Vivo

**Figure 4:**
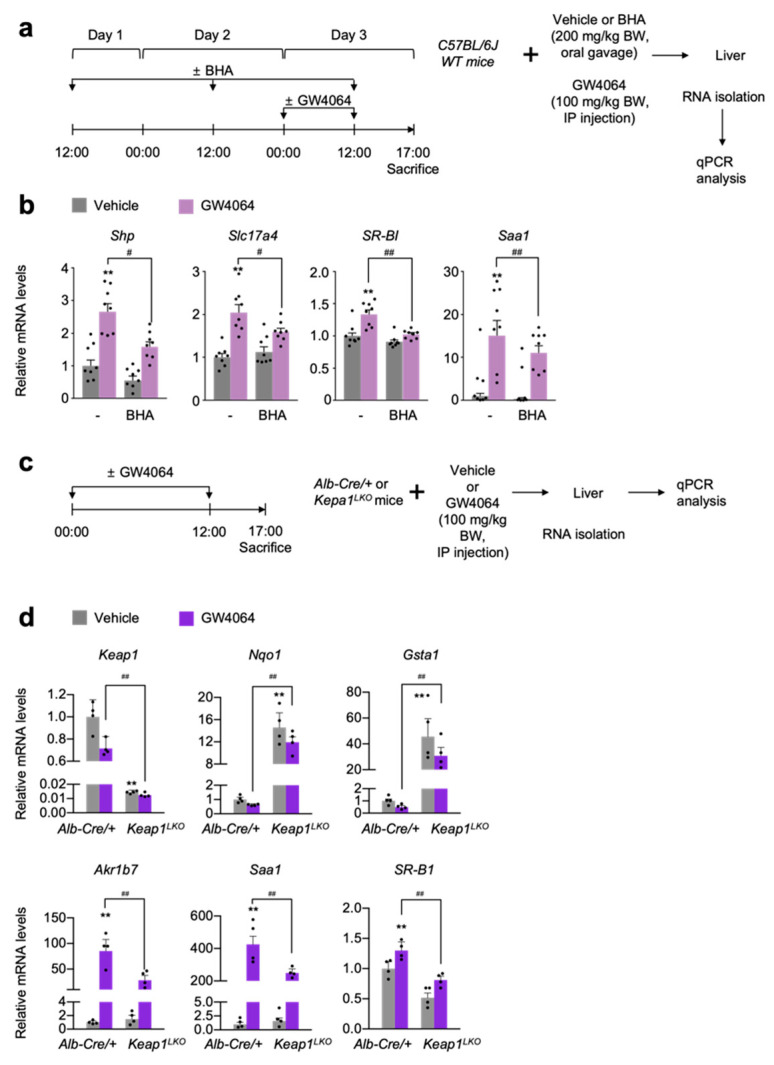
NRF2 activation is sufficient for suppressing a pharmacological transactivation of FXR in vivo. (**a**) A schematic diagram of an experimental procedure in mice. 8- to 9-week-old male *C57BL/6J* wild-type (*WT*) mice were orally gavaged with vehicle or butylated hydroxyanisole (BHA, 200 mg/kg BW) once a day for 3 days. During the last 24 h, all mice were intraperitoneally injected with vehicle (0.1% DMSO in 4:1 ratio of PEG 400 and Tween 80) or GW4064, a synthetic FXR agonist (100 mg/kg BW) twice a day (first injection: 00:00 a.m., second injection: 12:00 p.m.). 5 h after last treatment, all mice were sacrificed to collect livers whose total RNAs were prepared for qPCR analysis; (**b**) Hepatic expression levels of FXR target genes *Shp*, *Slc17a4*, *SR-B1*, *and Saa1* were determined shown in panel (**a**) by qPCR analysis. *n* = 8 per group, ** *p* < 0.01 vs. *WT* treated with vehicle. ^#^ *p* < 0.05, ^##^ *p* < 0.01; (**c**) A schematic diagram of an experimental procedure in mice. 8–9-week-old male *Alb-Cre/+* and *Keap1^LKO^* mice were intraperitoneally injected with vehicle (0.1% DMSO in 4:1 ratio of PEG 400 and Tween 80) or GW4064, a synthetic FXR agonist (100 mg/kg BW) twice a day. 5 h after the last injection, all mice were sacrificed to harvest livers whose total RNAs were isolated for qPCR analysis; (**d**) Hepatic expression levels of *Keap1*, NRF2 target genes (*Nqo1* and *Gsta1*), FXR target genes (*Akr1b7*, *Saa1*, and *SR-B1)* were determined shown in panel (**c**) by qPCR analysis. *n* = 4 per group; ** *p* < 0.01 vs. *Alb-Cre/+* treated with vehicle; ^##^ *p* < 0.01. Data represent mean ± s.e.m. and are plotted as fold change. Each dot indicates individual sample. Statistics by a two-tailed, unpaired Student *t*-test.

To validate these results in vivo, wild-type C57BL/6J mice were treated with butylated hydroxyanisole (BHA) [4], an NRF2 activator in an oral gavage manner for 3 days. At day 3, these mice were intraperitoneally injected with the vehicle or GW4064, as shown in Figure 4a. After that, the hepatic expression levels of FXR target genes *Shp*, *Slc17a4*, *SR-B1*, and *Saa1* were determined by qPCR analysis. As expected, GW4064 treatment increased the mRNA levels of these FXR target genes but these responses were significantly blunted in the livers of BHA-treated mice (Figure 4b). These results indicate that BHA-mediated NRF2 activation is sufficient for suppressing a pharmacological activation of FXR.

To genetically activate NRF2, we generated liver-specific *Keap1* knockout (*Keap1^LKO^*) mice by crossing female *Keap1^F/F^* mice with male *Alb-Cre* mice. Since floxed *Keap1* alleles in *Keap1^F/F^* mice turned out to be hypomorphic leading to the spontaneous activation of NRF2 [47,56], we used *Alb-Cre* control littermates as the control mice instead of *Keap1^F/F^* mice (Figure 4c). As expected, the livers of *Keap1^LKO^* mice showed an almost complete absence of *Keap1* expressions but marked increased mRNA levels of *Nqo1* and *Gasta1* compared with those of the control *Alb-Cre* mice (Figure 4d), indicating that a potent NRF2 activation occurs in the livers of *Keap1^LKO^* mice. Once again, elevated expression levels of hepatic FXR target genes *Akr1b7*, *Saa1*, and *SR-B1* in GW4064-treated *Alb-Cre* mice were significantly decreased in those of GW4064-treated *Keap1^LKO^* mice (Figure 4d). These data strongly suggest that genetical activation of NRF2 is also sufficient for suppressing pharmacological activation of FXR.

**Figure 5:**
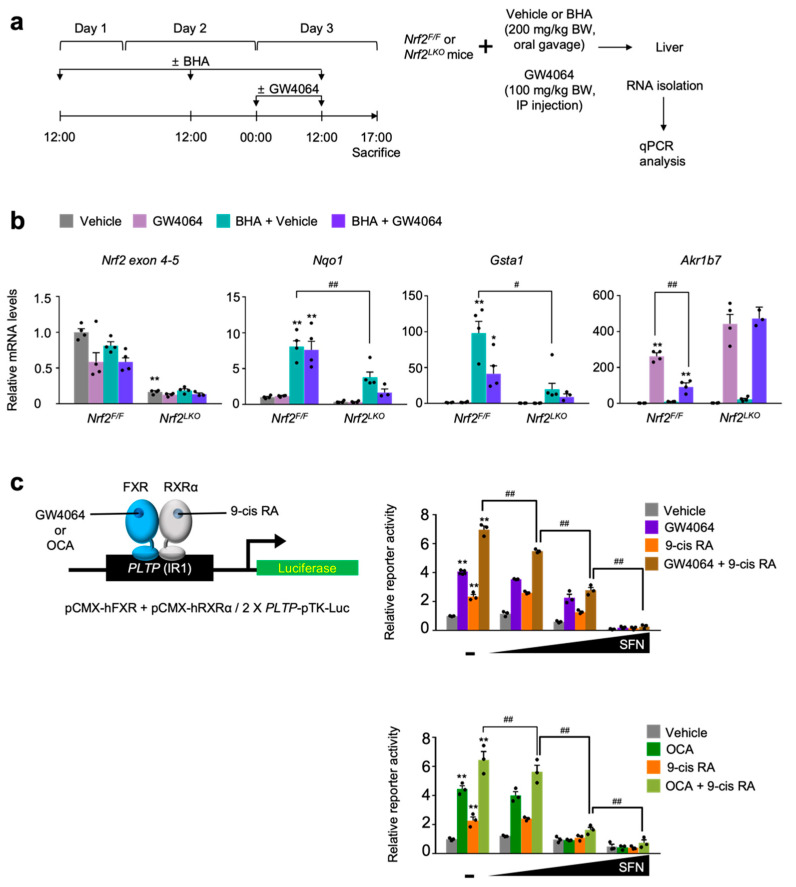
NRF2 is necessary for BHA-mediated suppression of a pharmacological transactivation of FXR. (**a**) A schematic diagram of an experimental procedure in mice. 8- to 9-week-old male *Nrf2^F/F^* and *Nrf2^LKO^* mice were orally gavaged with vehicle or butylated hydroxyanisole (BHA, 200 mg/kg BW) once a day for 3 days. During the last 24 h, all mice were intraperitoneally injected with vehicle (0.1% DMSO in 4:1 ratio of PEG 400 and Tween 80) or GW4064, a synthetic FXR agonist (100 mg/kg BW) twice a day (first injection: 00:00 a.m., second injection: 12:00 p.m.). 5 h after last treatment, all mice were sacrificed to collect livers whose total RNAs were prepared for qPCR analysis; (**b**) Hepatic expression levels of *Nrf2*, its target genes *Nqo1* and *Gsta1*, and FXR target gene *Akr1b7* were determined shown in panel (**a**) by qPCR analysis. *n* = 3–4 per group, ** *p* < 0.01 vs. *Nrf2^F/F^* treated with vehicle. ^#^ *p* < 0.05, ^##^ *p* < 0.01; (**c**) Cell-based reporter assays. AML12 cells were transiently transfected with a 2× *PLTP* luciferase reporter construct (2× *PLTP*-pTK-Luc), CMX-β-galactosidase, and expression plasmids of FXR and RXRα. After 24 h of transfection, cells were treated with either GW4064 (1 μM), 9-cis retinoic acid (9-cis RA, 1 μM), obeticholic acid (OCA, 10 μM) or both of GW4064 (or OCA) and 9-cis RA in the presence or absence of sulforaphane (SFN, 12.5, 25, or 50 μM) in a dose-dependent manner for 24 h. Vehicle is 0.1% DMSO; Normalized values (luciferase activity/β-galactosidase acidity) of vehicle-treated cells were set as fold 1; ** *p* < 0.01 vs. vehicle; ^#^ *p* < 0.05; ^##^ *p* < 0.01. Data represent mean ± s.e.m. and are plotted as fold change. Each dot indicates individual sample. Statistics by a two-tailed, unpaired Student *t*-test.

To genetically confirm whether compromised pharmacological activations of FXR mediated by BHA treatment depend on NRF2 proteins in vivo, we generated liver-specific *Nrf2* knockout (*Nrf2^LKO^*) mice by crossing female *Nrf2^F/F^* mice with male *Alb-Cre* mice. Control *Nrf2^F/F^* and the *Nrf2^LKO^* mice were then orally gavaged with BHA once a day for 3 days. At day 3, these mice were intraperitoneally treated with the vehicle or GW4064 as shown in Figure 5a. As conditional knockout mice, hepatic *Nrf2* mRNA levels were markedly reduced in the *Nrf2^LKO^* mice, confirming a genetic ablation of *Nrf2* gene in the liver (Figure 4b). Similar to the results shown in the wild-type C57BL/6J mice (Figure 4a,b), BHA treatment also robustly increased hepatic expression levels of the NRF2 target genes *Nqo1* and *Gasta1* in *Nrf2^F/F^* control littermates but these responses were significantly compromised in *Nrf2^LKO^* mice (Figure 4b). These results demonstrate that induction of *Nqo1* and *Gsta1* genes by BHA treatment largely depends on NRF2, and that BHA indeed activates NRF2 in mouse livers. Furthermore, GW4064 treatment in *Nrf2^F/F^* control littermates also highly induced hepatic expressions of the FXR target gene *Akr1b7* but this induction was significantly decreased in the BHA-treated *Nrf2^F/F^* counterparts. In contrast, this suppressed expression level of *Akr1b7* gene were completely lost in the BHA-treated *Nrf2^LKO^* mice, demonstrating that blunted pharmacological transactivation of FXR mediated by BHA treatment indeed requires NRF2 proteins (Figure 4b).

Finally, we decided to determine whether pharmacological activation of NRF2 has an effect on FXR transactivation upon treatment of its synthetic agonists, cell-based reporter assays were performed in AML12 cells transiently transfected with 2× *PLTP*-pTK reporter construct [57] and expression plasmids of FXR and RXRα. These cells were then treated with agonist ligands GW4064, 9-cis retinoic acid (9-cis RA), obeticholic acid (OCA), or a combination of GW4064 (or OCA) and 9-cis RA in the presence or absence of sulforaphane in a dose-dependent manner (Figure 5c). As shown in Figure 2 and Figure 3, increasing concentrations of sulforaphane gradually decreased FXR transactivation in response to its agonist ligands, indicating that NRF2 activation suppresses the pharmacological activation of FXR (Figure 5c).

## 4. Discussion

A previous study has shown that specific knockdown of core-autophagy genes *Atg5* or *Atg7* in AML12 cells markedly impedes the induction of β-hydroxybutyrate, a ketone body in response to Wy-14,643, a synthetic PPARα agonist ligand in both basal and oleate-treated conditions. Similar to this, both treatments of GW7647, a more potent synthetic PPARα agonist than Wy-14,643 and fasting-elevated serum β-hydroxybutyrate levels in control *Atg7^F/F^* mice, but both responses were significantly compromised in *Atg7^LKO^* mice [40]. All these data suggest that core autophagy-related genes responsible for maintaining a basal autophagy activity may affect PPARα functions particularly associated with fasting-associated metabolism. Based on these findings, we hypothesize that autophagy defects might also affect functionalities of other nuclear receptors including FXR.

In this study, we demonstrate that both autophagy inhibition and NRF2 activation are able to impair a pharmacological activation of the nuclear bile acid receptor FXR. For instance, the livers of *Atg7^LKO^* mice showed compromised inductions of FXR target genes *Shp*, *Slc17a4*, *Bsep*, and *SR-B1* in response to GW4064 treatment (Figure 1a,b). These findings are also recapitulated in cultured mouse hepatocyte-derived cell line AML12 cells. Bafilomycin A1, a known autophagy inhibitor via the inactivation of lysosomal v-ATPase, potently suppressed upregulation of FXR target genes *Shp*, *Akr1b7*, and *Slc17a4* in the presence of GW4064 or obeticholic acid (OCA) (Figure 1c–e). These data suggest that either a genetic ablation of a core-autophagy gene *Atg7* or a pharmacological inhibition of autophagy profoundly affects the pharmacologically-activated transcriptional programs of FXR.

Previously, it has been shown that genetic ablation of *Atg7* robustly increases protein levels of p62, a ubiquitin-binding protein, which leads to NRF2 activation via its dissociation from KEAP1, an adaptor protein for Cullin3-based E3 ubiquitin ligase. Liberated NRF2 is then translocated into the nucleus where it binds to antioxidant response element (ARE) together with small Maf (sMaf) proteins, resulting in the induction of phase II cytoprotective genes [2,51,52]. Based on these findings, we confirmed hepatic NRF2 activation in our *Atg7^LKO^* mice. Consistently, we observed markedly increased *Nqo1* mRNA levels in the livers of *Atg7^LKO^* mice but not in those of control *Atg7^F/F^* mice (Figure S1a). Consistent with this in vivo study, dose-dependent treatments of bafilomycin A1 in AML12 cells also significantly induced expression of *Hmox1* gene but gradually decreased *Nqo1* mRNA levels (Figure S1b,c). Since *Nqo1* and *Hmox1* are well-known NRF2 target genes, we suspect that they have different expression kinetics upon bafilomycin A1-mediated NRF2 activation in AML12 cells. Taken together, these results indicate that autophagy inhibition robustly activates NRF2 in mouse livers and mouse hepatocyte-derived cell line AML12 cells.

To examine whether a direct pharmacological activation of NRF2 affects FXR functionalities, AML12 cells were co-treated with small molecules of NRF2 activators and synthetic FXR agonists. Both dose- and time-dependent treatments of sulforaphane or dimethyl fumarate remarkably induced *Nqo1* and *Hmox1* expressions but markedly impeded the induction of FXR target genes *Shp*, *Ark1b7*, and *Slc17a4* in response to the treatment of GW4064 (Figure 2 and Figure 3) or OCA (Figures S2 and S3). Similarly, a 3 day treatment of BHA, another known NRF2 activator in wild-type C57BL/6J mice also significantly blunted the induction of hepatic FXR target genes *Shp*, *Slc17a4*, *SR-B1*, *and Saa1* in response to the treatment of GW4064 (Figure 4a,b). In addition, to investigate whether a genetic activation of NRF2 also affects FXR functionalities, *Alb-Cre/+* and *Keap1^LKO^* mice were intraperitoneally treated with the vehicle or GW4064 (Figure 4c). As expected, [47], both *Nqo1* and *Gasta1* of NRF2 target genes were strongly induced in the livers of *Keap1^LKO^* mice compared with those of *Alb-Cre/+* mice (Figure 4d), confirming that *Keap1* ablation leads to NRF2 activation. Consistent with the results of BHA experiments (Figure 4a,b), NRF2 activation in *Keap1^LKO^* mice significantly impaired the induction of FXR target genes *Akr1b7*, *Saa1*, and *SR-B1* in response to the treatment of GW4064 (Figure 4d). These findings suggest that NRF2 activation is sufficient to inhibit the pharmacological activation of FXR.

To examine whether impairment of pharmacological FXR transactivation upon BHA treatment depends on NRF2, *Nrf2^F/F^* and *Nrf2^LKO^* mice were co-treated with both BHA and GW4064 (Figure 5a). As expected, the 3 day treatment of BHA in *Nrf2^F/F^* control littermates markedly increased NRF2 target genes *Nqo1* and *Gasta1* in the livers but these responses were significantly decreased in *Nrf2^LKO^* mice, confirming that BHA indeed activates NRF2 in our experimental conditions (Figure 5b). In accordance with previous studies, GW4064 also potently upregulates hepatic FXR target gene *Akr1b7* in control *Nrf2^F/F^* mice but this response was significantly compromised in BHA-treated *Nrf2^F/F^* mice. However, to our surprise, this blunted response was completely lost in BHA-treated *Nrf2^LKO^* mice (Figure 5b). These results suggest that NRF2 is indeed necessary and sufficient for the impairment of pharmacological FXR activation upon BHA treatment.

Finally, to assess whether sulforaphane-mediated NRF2 activation affects FXR transactivation in cell-based reporter assays, AML12 cells were transiently transfected with a 2× *PLTP*-pTK-Luc reporter construct containing two FXR response elements. At the same time, these cells were co-transfected with expression plasmids encoding *Fxr* and *Rxrα*. Dose-dependent treatments of sulforaphane minimally affect luciferase activities except its highest concentration (50 μM) in vehicle-treated cells. In contrast, agonist responses for FXR, RXRα or both upon treatment of their ligands were gradually decreased by increasing concentrations of sulforaphane, indicating that NRF2 activation is sufficient to compromise FXR transactivation at least in cell-based reporter assays.

The Komatsu and Panasyuk laboratories have also reported similar results regarding the impairment of PPARα transactivation by autophagy defects, although their molecular mechanisms are different from ours [58,59]. They have found that NCoR1 corepressor protein is degraded by autophagy in the fasted state and that this corepressor is remarkably accumulated in autophagy defective hepatocytes obtained from *Atg5*, *Atg7* or *Vps15* conditional knockout mice. These increased NCoR1 levels are accumulated in the nucleus where they tend to be recruited to PPARα binding sites, which lead to suppression of its target gene expression. This NCoR1 dependency has been demonstrated in liver-specific *Atg7* and *Ncor1* double knockout mice, reversing compromised transactivation of PPARα. In terms of detailed molecular mechanism by which autophagy defects impede PPARα signaling, we speculate that there are many more mechanisms yet to be discovered. Additionally, the Yin laboratory has also reported that autophagy defects in *Atg5^LKO^* or *Atg7^LKO^* mice compromise FXR functionality and lead to cholestatic liver injury [60].

In the future research, to address whether the impairment of a pharmacological activation of FXR in *Atg7^LKO^* mice is dependent on NRF2 activation, it is necessary to generate liver-specific *Atg7* and *Nrf2* double knockout (*Atg7^LKO^*; *Nrf2^LKO^*) mice. Nevertheless, it is of interest to note that increased liver damages and sizes observed in *Atg7^LKO^* mice were markedly decreased in either *Atg7^F/F^*; *Mx1*; *p62^−/−^* [51] or *Atg7^F/F^*; *Mx1*; *Nrf2^−/−^* mice [52]. In contrast, *Keap1^LKO^*; *Atg7^LKO^* mice showed even further increased alanine aminotransferase (ALT) and aspartate aminotransferase (AST) levels and bigger liver sizes than those of *Atg7^LKO^* mice [52]. These data suggest that NRF2 activation in autophagy defective conditions might be deleterious to maintaining liver homeostasis via the impediment of certain transcriptional programs executed by nuclear receptor signaling including PPARα and FXR. Moreover, it is intriguing to examine whether the blunted pharmacological activation of FXR in *Keap1^LKO^* mice is genetically dependent on NRF2, which might be tested using liver-specific *Nrf2* and *Keap1* double knockout (*Nrf2^LKO^*; *Keap1^LKO^*) mice to be generated.

Further investigations are also needed to define the detailed molecular mechanisms by which nuclear NRF2 proteins interfere with the functions of the nuclear receptor FXR. These potential mechanisms include a direct NRF2-FXR (or RXR) interaction, squelching coactivators, interfering with coactivator recruitment, promoting corepressor recruitment, inhibiting FXR binding to the genome, and so forth.

## 5. Conclusions

**Figure 6:**
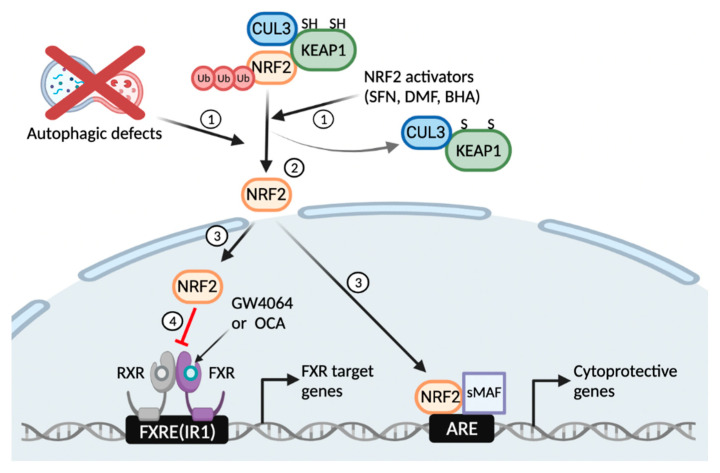
Working model of autophagy defects or NRF2 activators that blunt a pharmacological transactivation of the nuclear bile acid receptor FXR. Similar to NRF2 activators, autophagy inhibition by pharmacologic or genetic manipulations liberates NRF2 from KEAP1-CUL3-mediated proteasomal degradation (1, 2); This event leads to the nuclear translocation of NRF2 from the cytoplasm, where it acts as a potent antioxidant transcription factor regulating expression of many phase 2 cytoprotective genes (3); On the other hand, the nuclear NRF2 interferes with a pharmacological transactivation of FXR, resulting in impairment of its target gene expression (4). FXRE, FXR response element; IR1, inverted repeat 1; ARE, antioxidant response element. This schematic diagram was created in BioRender.com (accessed on 12 January 2022).

In summary, our results reveal a critical function of basal autophagy for the pharmacological activation of the nuclear bile acid receptor FXR. Autophagy defects or NRF2 activators induce the translocation of NRF2 from the cytoplasm to the nucleus where it regulates the expression of phase II cytoprotective genes. On the other hand, it seems likely that these nuclear NRF2 proteins are able to interfere with functions of other transcription factors and nuclear receptors including FXR via numerous mechanisms (Figure 6). Thus, this study suggests unexpected roles of basal autophagy flux or NRF2 activation that has a profound impact on the regulation of the nuclear receptor FXR signaling, which could be harnessed to treat bile acid-associated metabolic diseases.

## Data Availability

Data contained within this article and the supplementary.

## Supplementary Materials

The following supporting information can be downloaded at: https://www.mdpi.com/article/10.3390/antiox11020370/s1, Figure S1: NRF2 activation in response to autophagy inhibition; Figure S2: DMF-mediated NRF2 activation in a dose-dependent manner impairs a pharmacological activation of FXR; Figure S3: DMF-mediated NRF2 activation in a time-dependent manner impairs a pharmacological activation of FXR; Table S1: Mouse primer sequences for qPCR analysis.
