# Supplementary material for: NRF2 Activation in Autophagy Defects Suppresses a Pharmacological Transactivation of the Nuclear Receptor FXR
Source: Antioxidants (Basel). 2022 Feb 12;11(2):370. doi: 10.3390/antiox11020370 (PMC8868494; doi:10.3390/antiox11020370)
Supplement: Supplementary file 1 [file antioxidants-11-00370-s001.zip › antioxidants-1575387-supplementary.pdf]

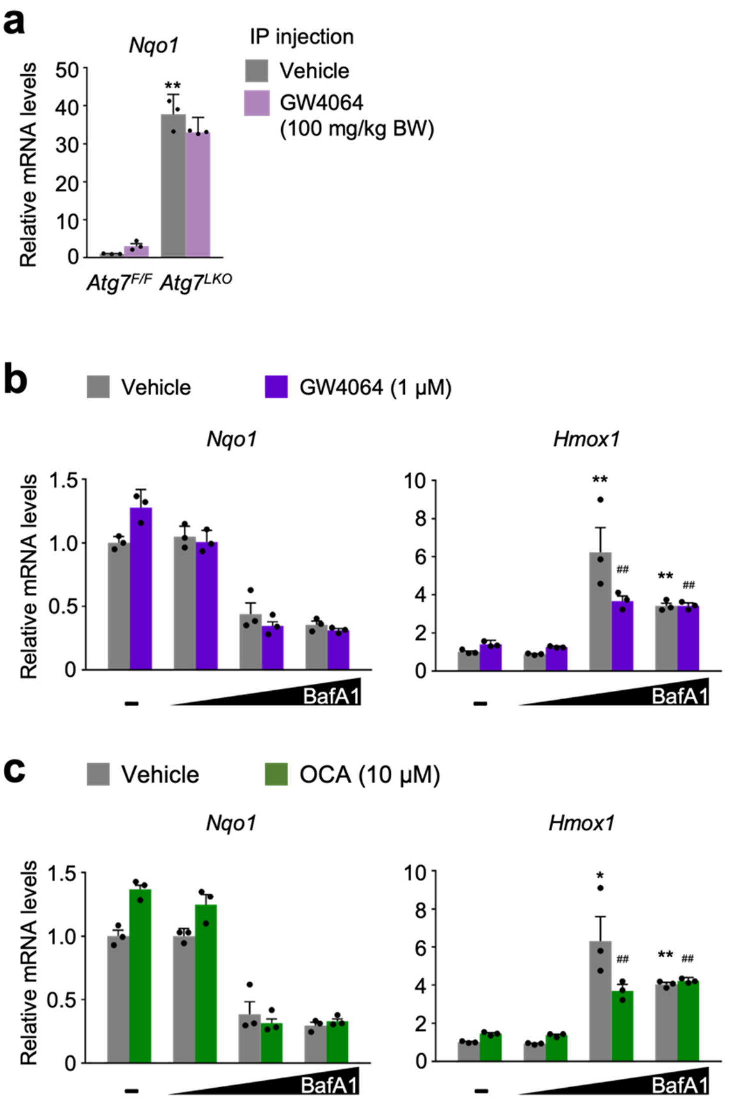

Figure S1. Upregulation of NRF2 target genes in response to autophagy inhibition. Male *Alb-Cre* mice were crossed with female *Atg7*$^{F/F}$ mice to generate liver-specific *Atg7* knockout (*Atg7*^{LKO}) mice as well as control littermate *Atg7*$^{F/F}$ mice. (**a**) Hepatic expression levels of *Nqo1*, an NRF2 target gene were determined in *Atg7*$^{F/F}$ and *Atg7*^{LKO} mice shown in Figure 1a by qPCR analysis. n = 4 per group, $^{**}p < 0.01$ vs *Atg7*$^{F/F}$ mice treated with vehicle. $^{\#\#}p < 0.01$. Data represent mean ± s.e.m. and are plotted as fold change. Each dot indicates individual mouse. (b,**c**) Expression levels of NRF2 target genes *Nqo1* and *Hmox1* were determined in AML12 cells shown in Figure 1c by qPCR analysis. n = 3 per group, $^{**}p < 0.01$ vs AML12 cells treated with vehicle. $^{\#\#}p < 0.01$ vs AML12 cells treated with GW7647. Data represent mean ± s.e.m. and are plotted as fold change. Each dot indicates individual sample. Statistics by a two-tailed, unpaired Student *t*-test. IP injection, intraperitoneal injection; BW, body weight; BafA1, bafilomycin A1; OCA, obeticholic acid.

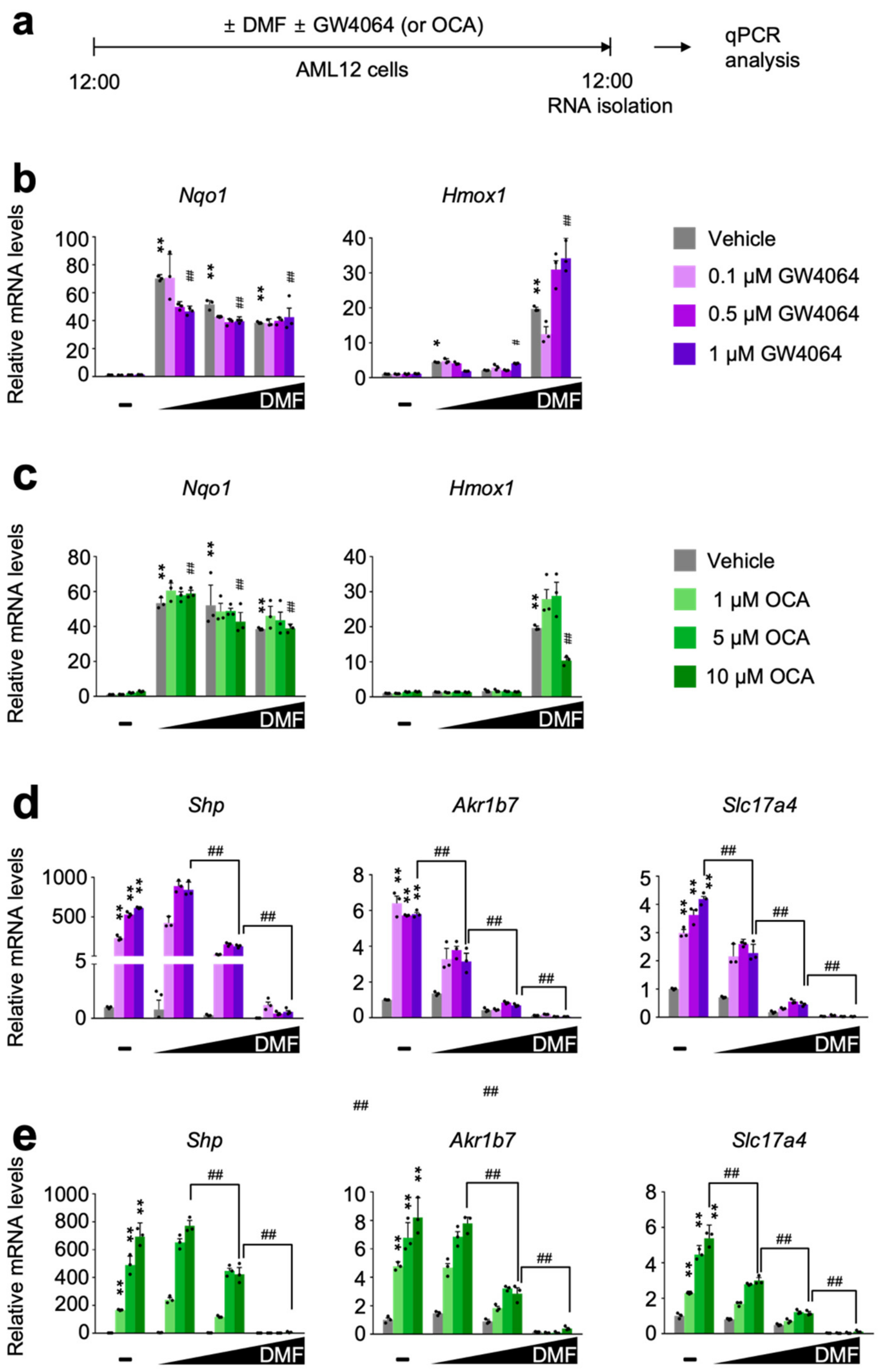

Figure S2. DMF-mediated NRF2 activation in a dose-dependent manner impairs a pharmacologic FXR transactivation. (**a**) A schematic diagram of an experimental procedure in AML12 cells. AML12 cells were treated with dimethylfumarate (DMF, 50, 75, or 100 μM), a known NRF2 activator in a dose-dependent manner in the absence or presence of synthetic FXR agonists (GW4064: 0.1, 0.5 or 1 μM; OCA: 1, 5 or 10 μM) for 24 hr. Vehicle is 0.1% DMSO. Total RNAs from these cells were prepared to perform qPCR analysis. (**b**,**c**) Expression levels of NRF2 target genes *Nqo1* and *Hmox1* were determined in AML12 cells shown in panel **a** by qPCR analysis. n = 3 per group. $^{**}p < 0.01$ vs. AML12 cells treated with vehicle. $^{\#\#}p < 0.01$ vs. AML12 cells treated with 1 μM GW4064. (**d**,**e**) Expression levels of FXR target genes *Shp*, *Ark1b7*, and *Slc17a4* were determined in AML12 cells shown in panel **a** by qPCR analysis. n = 3 per group, $^{*}p < 0.05$, $^{**}p < 0.01$ vs. AML12 cells treated with vehicle. $^{\#\#}p < 0.01$. Data represent mean ± s.e.m. and are plotted as fold change. Each dot indicates individual sample. Statistics by a two-tailed, unpaired Student *t*-test. DMF, dimethylfumarate; OCA, obeticholic acid.

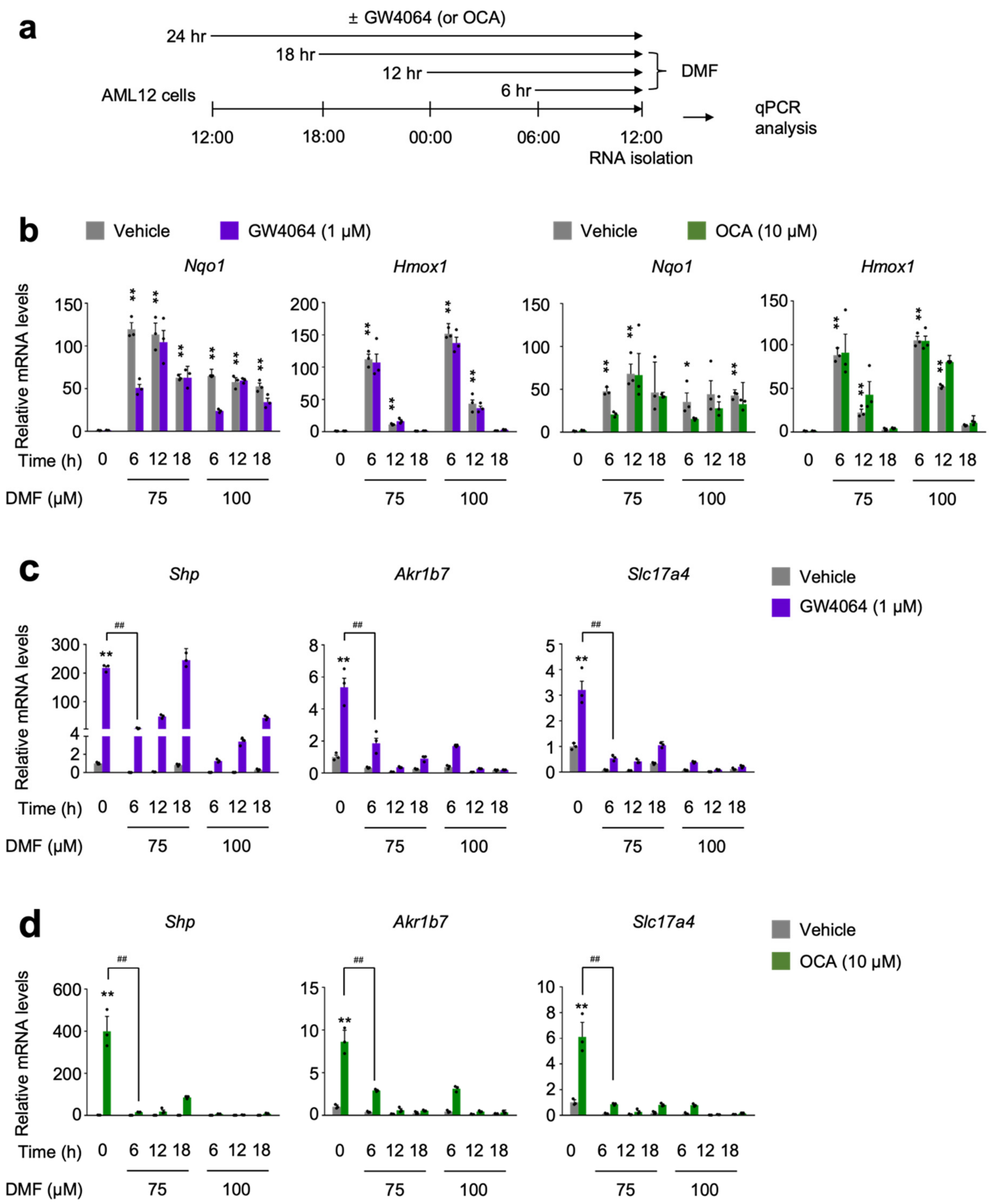

Figure S3. DMF-mediated NRF2 activation in a time-dependent manner impairs a pharmacologic FXR transactivation. (**a**) A schematic diagram of an experimental procedure in AML12 cells. AML12 cells were treated with either 75 μM or 100 μM of dimethylfumarate (DMF), a known NRF2 activator in a time-dependent manner (6 h, 12 h or 18 h) in the absence or presence of synthetic FXR agonists (1 μM of GW4064; 10 μM of OCA) for 24 h. Vehicle is 0.1% DMSO. Total RNAs from these cells were prepared to perform qPCR analysis. (**b**) Expression levels of NRF2 target genes *Nqo1* and *Hmox1* were determined in AML12 cells shown in panel **a** by qPCR analysis. *$p < 0.05$, **$p < 0.01$ vs. AML12 cells treated with vehicle. ##$p < 0.01$ vs. AML12 cells treated with either GW4064 or OCA. (**c**,**d**) Expression levels of FXR target genes *Shp*, *Akr1b7*, and *Slc17a4* were determined in AML12 cells shown in panel **a** by qPCR analysis. n = 3 per group, *$p < 0.05$, **$p < 0.01$ vs. AML12 cells treated with vehicle. ##p < 0.01. Data represent mean ± s.e.m. and are plotted as fold change. Each dot indicates individual sample. Statistics by a two-tailed, unpaired Student *t*-test. DMF, dimethylfumarate; OCA, obeticholic acid.

Table S1. Mouse primer sequences for qPCR analysis

Gene	Sequences
Atg7 exon 14-15 Fwd	CTAATGGACACCAGGGAGAG
Atg7 exon 14-15 Rvs	CATGTCTCATGACAACAAAGGT
Pdk4 Fwd	GTCGAGCATCAAGAAAACCGTCC
Pdk4 Rvs	TGTGATGCCCTTCAGGAAGGAG
Acot3 Fwd	ACTACGAGGACCTCCCTAAGGA
Acot3 Rvs	CATGGCAAAGCCAAGTTCACCC
Ucp2 Fwd	TAAAGGTCCGCTTCCAGGCTCA
Ucp2 Rvs	ACGGGCAACATTGGGAGAAGTC
Acot2 Fwd	AAGAAGCCGTGAACTACCTGCG
Acot2 Rvs	TGTGATGCCCTTCAGGAAGGAG
Cidec Fwd	TCGGAAGGTTCGCAAAGGCATC
Cidec Rvs	CTCCACGATTGTGCCATCTTCC
Acox1 Fwd	GCCATTCGATACAGTGCTGTGAG
Acox1 Rvs	CCGAGAAAGTGGAAGGCATAGG
Fgf21 Fwd	ATCAGGGAGGATGGAACAGTGG
Fgf21 Rvs	AGCTCCATCTGGCTGTTGGCAA
Nrf2 exon 4-5 Fwd	TTAAGCAGCATAGAGCAGGA
Nrf2 exon 4-5 Rvs	TTCTGTCAGTGTGGCTTCTG
Nqo1 Fwd	GCCGAACACAAGAAGCTGGAAG
Nqo1 Rvs	GGCAAATCCTGCTACGAGCACT
Hmox-1 Fwd	CACTCTGGAGATGACACCTGAG
Hmox-1 Rvs	GTGTTCCTCTGTCAGCATCACC
Gstp1 Fwd	TGGAAGGAGGAGGTGGTTACCA
Gstp1 Rvs	GGTAAAGGGTGAGGTCTCCATC
36B4 Fwd	CAACCCAGCTCTGGAGAAAC
36B4 Rvs	CCAACAGCATATCCCGAATC
Keap1 exon 4-5 Fwd	ATCCAGAGAGGAATGAGTGGCG
Keap1 exon 4-5 Rvs	TCAACTGGTCCTGCCCATCGTA
Gsta1 Fwd	GTGCCTTGGCAAAAGATAGGACC
Gsta1 Rvs	CTTCCAGTAGGTGGATGTCCAC
